# The Characteristic of Biofilm Formation in ESBL-Producing *K. pneumoniae* Isolates

**DOI:** 10.1155/2024/1802115

**Published:** 2024-09-19

**Authors:** Xiaofang Gao, Haili Wang, Zhijuan Wu, Pan Sun, Wei Yu, Donghua Chen, Yuhua Mao, Lili Fang, Jia Qian, Li Li, Qian Peng, Yanping Han

**Affiliations:** ^1^ Jiading District Center for Disease Control and Prevention, Shanghai 201800, China; ^2^ Central Medical Branch of PLA General Hospital, Beijing 100120, China; ^3^ Department of Prevention and Healthcare Community Health Service Center of Waigang Town, Jiading, Shanghai 201806, China; ^4^ Department of Infectious Diseases North Campus of Ruijin Hospital, Shanghai 201800, China; ^5^ State Key Laboratory of Pathogen and Biosecurity Academy of Military Medical Sciences, Beijing 100071, China

## Abstract

*Klebsiella pneumoniae* is a pathogen that commonly causes hospital-acquired infections. Bacterial biofilms are structured bacterial communities that adhere to the surface of objects or biological tissues. In this study, we investigated the genome homology and biofilm formation capacity of ESBL-producing *K. pneumoniae*. Thirty ESBL-producing *K. pneumoniae* isolates from 25 inpatients at Ruijin Hospital, Shanghai, were subjected to pulsed-field gel electrophoresis (PFGE) to estimate genomic relatedness. Based on the chromosomal DNA patterns we obtained, we identified 21 PFGE profiles from the 30 isolates, eight of which had high homology indicating that they may have genetic relationships and/or potential clonal advantages within the hospital. Approximately 84% (21/25) of the clinical patients had a history of surgery, urinary tract catheterization, and/or arteriovenous intubation, all of which may have increased the risk for nosocomial infections. Biofilms were observed in 73% (22/30) of the isolates and that strains did not express type 3 fimbriae did not have biofilm formation capacity. Above findings indicated that a high percentage of ESBL-producing *K. pneumoniae* isolates formed biofilms *in vitro* and even though two strains with cut-off of PFGE reached 100% similarity, they generated biofilms differently. Besides, the variability in biofilm formation ability may be correlated with the expression of type 3 fimbriae. Thus, we next screened four ESBL-producing *K. pneumoniae* isolates (Kpn5, Kpn7, Kpn11, and Kpn16) with high homology and significant differences in biofilm formation using PFGE molecular typing, colony morphology, and crystal violet tests. Kpn7 and Kpn16 had stronger biofilm formation abilities compared with Kpn5 and Kpn11. The ability of above four ESBL-producing *K. pneumoniae* isolates to agglutinate in a mannose-resistant manner or in a mannose-sensitive manner, as well as RNA sequencing-based transcriptome results, showed that type 3 fimbriae play a significant role in biofilm formation. In contrast, type 1 fimbriae were downregulated during biofilm formation. Further research is needed to fully understand the regulatory mechanisms which underlie these processes.

## 1. Introduction

*K. pneumoniae* is a Gram-negative opportunistic pathogen that is widespread in clinical and nonclinical settings [1]. Clinically, ESBL-producing *K. pneumoniae* mainly causes nosocomial infections in hospitalized patients, including respiratory, urinary tract, blood, and wound infections [2, 3]. Additionally, antibiotic resistance in *K. pneumoniae* and other bacterial pathogens continues to be a growing public health concern. ESBL-producing *K. pneumoniae* are increasingly common and are difficult to treat. The extracellular matrices that makeup biofilms can trap antibiotics and prevent them from reaching bacterial cells. Additionally, bacteria have slowed metabolic rates within the biofilms, which makes them less susceptible to antibiotics that target rapidly dividing cells [4]. Biofilm formation increases bacterial density and provides favorable conditions for horizontal gene transfer, which allows for more frequent transmission of resistance genes between different bacterial species [5]. Medical devices are another important driver of bacterial infection, as they are susceptible to bacterial contamination both before and after implantation and can be growth surfaces for biofilms [6–9]. Thus, studying the mechanisms underlying ESBL-producing *K. pneumoniae* biofilm formation is of critical importance.

Biofilm formation in *K. pneumoniae* appears to be influenced by a variety of factors, including fimbriae formation, polysaccharides, and environmental conditions. Most *K. pneumoniae* produce type 1 and type 3 fimbriae. Type 1 fimbriae are short, hair-like structures that play a role in host tissue adhesion. Type 3 fimbriae, in contrast, are longer and more flexible, and are strongly associated with biofilm formation and bacterial aggregation. Type 1 fimbriae are encoded by the *fim* gene cluster, with the *fimA* gene encoding the major subunit and the *fimH* gene encoding the adduct located at the tip of fimbriae [10]. Type 1 fimbriae expression is associated with refractory urinary tract infections. Type 3 fimbriae are encoded by the *mrkABCDF* operon and mediate biofilm formation both *in vitro* and in vivo [11]. These fimbriae originate from the grana on the internal side of the bacterial membrane. Additionally, *K. pneumoniae* membrane protein integrity expression plays a crucial role in fimbriae structural stability, but the specific proteins and regulatory targets need to be further explored. The aim of this study is to evaluate the biofilm formation abilities of ESBL-producing *K. pneumoniae* isolates. Our findings may promote a more in-depth understanding of the regulatory process underlying fimbriae expression and thus lay the foundation for the therapeutic prevention of biofilm formation.

## 2. Materials and Methods

### 2.1. Bacterial Cultures

Thirty ESBL-producing *K. pneumoniae* isolates used in this study were provided by the Department of Infectious Diseases, North Campus of Ruijin Hospital, Shanghai during 2020-2021. All strains were identified and analyzed by VITEK2 Compact system (bioMérieux VITEK, USA). For bacterial cultures, 25 *μ*L of clinically isolated ESBL-producing *K.pneumoniae* glycerol stocks were inoculated into 5 mL Luria–Bertani (LB) broth and cultured overnight in a shaking incubator at 37°C and 200 rpm to the middle exponential phase (OD600 ≈ 1.0). Cultures were then finished, collected and reserved for the next experiment.

### 2.2. Antibiotic Susceptibility Testing

All strains were identified and analyzed by the VITEK2 Compact system (bioMérieux VITEK, USA) and drug susceptibility analysis system. The production of strain *β*-lactamase (ESBL) was tested by broth microdilution method. The specific drugs are as follows: cefepime (CPM), ceftazidime (CAZ), cefotaxime (CTX), aztreonam (AZT), ciprofloxacin (CIP), ampicillin-sulbactam (AMS), imipenem (IMI), meropenem (MEM) and piperacillin-tazobactam (PTZ), sulfamethoxazole (SXT), gentamycin (GEN), and levofloxacin (LEF). Specific operational details and result interpretation were conducted according to CLSI 2019 relevant standards [12].

### 2.3. Pulsed-Field Gel Electrophoresis (PFGE)

Our ESBL-producing *K. pneumoniae* strains were isolated and purified on Columbia blood plates and then prepared into a bacterial suspension with a turbidity of 0.48–0.52 using CSB (100 mM Tris, 100 mM EDTA, pH 8.0). The chromosomes of the 30 ESBL-producing *K. pneumoniae* isolates were digested with the restriction enzyme *XbaI* and electrophoresis was performed on the CHEF Mapper XA System (Bio-Rad). When completed, gels were stained with Gelred (Biotium) to display characteristic patterns under the Gel Doc UV light (Bio-Rad). The maps of PFGE were analyzed using Bionumerics 6.0 software and calculated by the Dice coefficients. The dendrogram was performed through the unweighted-pair group method with average linkages (UPGMA) [13].

### 2.4. Evaluation of Biofilm Formation Ability

We employed two methods: crystal violet stains and the rugose colony morphology assay to evaluate ESBL-producing *K. pneumoniae* isolate biofilm formation [14]. For crystal violet stained biofilms, we completed routine bacterial cultures and cultures were separately diluted (1 : 100) in 200 *μ*L LB medium in 96-well cell culture plate for 48 h. After incubation, the planktonic bacteria were moved and each aperture was washed three times and incubated at 60°C for 20 min to fix the biofilms. The apertures were then stained with 0.1% crystal violet for 20 min. The relative quantity of biofilm formation was represented by OD_570_/OD_600_ values. For the rugose colony morphology assay, 2 *μ*L of glycerol stocks were spotted onto LB plates, which were dried at 37°C for 24−48 h and then equilibrated at room temperature for 2 h. After bacterial plaque maturation, colony surfaces either showed visible biofilm folds or did not, allowing for analysis of differences in biofilm fold formation capabilities among different strains. Paired Student's *t*-test was performed to determine statistically significant differences and the OD_570_/OD_600_ values were calculated to indicate the relative biofilm formation. *P* ≤ 0.01 was considered to indicate statistical significance.

### 2.5. Hydrogenated Sheep Red Blood Cell and Guinea Pig Red Blood Cell Agglutination Assays

After routine bacterial cultures and cultures were separated 100 *μ*L of the four experimental strains to 24-well plate covers and then added 50 *μ*L of 6% hydrolyzed sheep erythrocytes and 50 *μ*L of 5% D-mannose solution.

100 *μ*L of PBS solution was used as a negative control. We also completed identical experiments where 6% hydroformylation sheep erythrocytes were substituted with 6% guinea pig erythrocytes. For bacterial agglutination assays, the representative data from at least three independent biological replicates were shown. For bacterial erythrocyte agglutination titer ratio, Paired Student's *t*-test was performed to determine statistically significant differences and *P* ≤ 0.01 was considered to indicate statistical significance.

### 2.6. RNA Extraction and RNA-Seq

We cultured ESBL-producing *K. pneumoniae* through routine way. After incubation, we added RNA protectant (RNaseZap) to bacterial cultures, and then concentrated these bacterial broths using centrifugation. Total RNA was extracted following the PureLink™ RNA Mini Kit (Thermo Scientific) experimental protocols. RNA quantity and concentration were determined using NanoDrop 2000 spectrophotometry (Thermo Scientific). Finally, total RNA results were used to construct a cDNA library, and high-throughput sequencing was performed as previously described. The Fragments per Kilobase per Million (FPKM) value was used to measure the amount of ESBL-producing *K. pneumoniae* single gene transcripts and to calculate the relative differences in gene expression between different strains.

### 2.7. Quantitative RT-PCR (qRT-PCR)

Real-time quantitative PCR refers to the real-time analysis and detection of cumulative amplified PCR products during the reaction. To verify the differentially expressed genes suggested by RNA-seq, we used qRT-PCR on the Light Cycler 480 II (Roche, USA), with 16 S rRNA as the internal standard to standardize the expression of all sRNA candidates (Table 1). The primer we drew the 16 S rRNA gene relative standard curve, calculated the relative template quantity of the genes, and analyzed the results.

### 2.8. Statistical Analysis

For all quantity bacterial assays, the representative data were from at least three independent biological replicates. The data were analyzed with *SPSS 20.0* and Paired Student's *t*-test was performed to determine statistically significant differences and *P* ≤ 0.01 was considered to indicate statistical significance.

## 3. Results

### 3.1. Clinical Characteristics of ESBL-Producing *K. pneumoniae* Isolates in This Study

In this study, 30 clinical ESBL-producing *K. pneumoniae* isolates were collected from 25 patients aged an average of 62 years. The average duration of hospitalization for the patients was 42 days. Approximately 84% (21/25) of these patients had a history of surgery, urinary tract catheterization, and/or arteriovenous intubation, all of which were risk factors for nosocomial infections such as *K. pneumoniae* (Table 2). Besides, the strains in this study were multiple resistant bacteria which were simultaneously insensitive to at least 10 antimicrobial drugs (Table 3).

### 3.2. The Evaluation Biofilm Ability of ESBL-Producing *K. pneumoniae* Isolates

**Figure 1:**
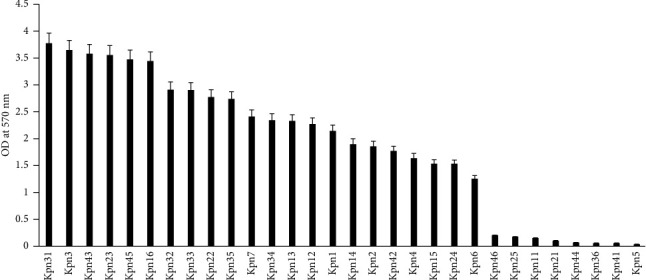
Biofilm formation in ESBL-producing *K. pneumoniae* strains. Biofilm formation by 30 ESBL-producing *K. pneumoniae* isolates was monitored with crystal violet staining and quantified with OD_570_ values. OD_570_ > 1 were classified as biofilm-forming strains, and OD_570_ < 1 were classified as nonbiofilm-forming strains.

The classification of biofilm-producing strains was based on OD_570_ values. Strains with OD_570_ < 1 were considered to be non-biofilm formers, and strains with OD_570_ ≥ 1 were considered to be biofilm formers. Among the ESBL-producing *K. pneumoniae* isolates, 73% (22/30) formed biofilms (Figure 1). In addition, specific detection of type 3 fimbriae expression in ESBL-producing *K. pneumoniae* isolates via mannose-resistant agglutination of acid-sheep RBCs indicated that high percentages of ESBL-producing *K. pneumoniae* isolates formed biofilms *in vitro*, but that strains did not express type 3 fimbriae could not form biofilms.

### 3.3. Homology Analysis of ESBL-Producing *K. pneumoniae* Isolates

**Figure 2:**
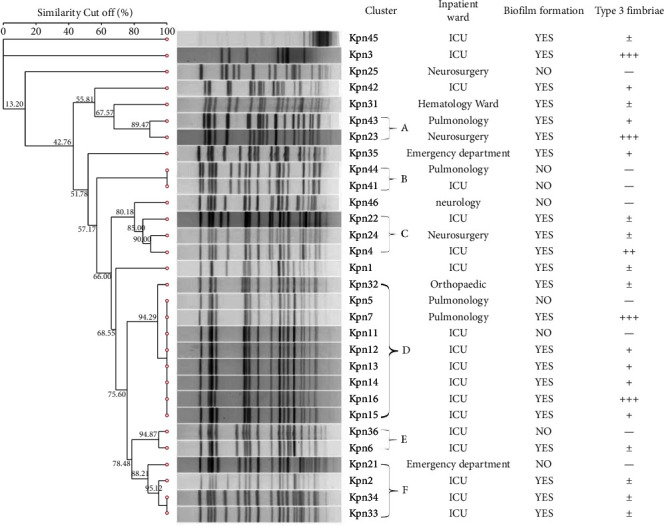
PFGE cluster analysis results of ESBL-producing *K. pneumoniae* strains. Dendrogram representing the genetic relationship between 30 ESBL-producing *K. pneumoniae* isolates after restriction with the *XbaI* enzyme. The gene cluster was formed on the dendrogram with a similarity cut off between 85% and 100% of which 22 PFGE stripe of ESBL-producing *K. pneumoniae* contained 6 gene clusters (A–F).

After PFGE cluster analysis, based on drown dendrogram 22 clusters with more than 85% similarity cut-off were found among 30 isolates. Similarity from 85% to 100% on the dendrogram form a gene cluster (A–F) among which gene cluster *D* (including Kpn5/7/11/16) had the highest percentage of 40% (9/22) and may have signal clonal advantage. By considering the distinct pulsotypes obtained in the current study it seems that the genetic heterogeneity of ESBL-producing *K. pneumoniae* is more conserved in the North of Ruijin Hospital (Figure 2). Quantitative analysis of biofilm formation using 0.1% crystal violet staining showed significant differences in biofilm formation between the Kpn5 and Kpn7 strains, as well as between the Kpn11 and Kpn16 strains. To reduce interference factors and narrow the screening scope for potential biofilm formation target genes, four clinical ESBL-producing *K. pneumoniae* isolates with high homology and significant differences in biofilm formation were selected for further analysis.

### 3.4. Differential Evaluation of Biofilm Formation Amongst Four ESBL-Producing *K. pneumoniae*

**Figure 3:**
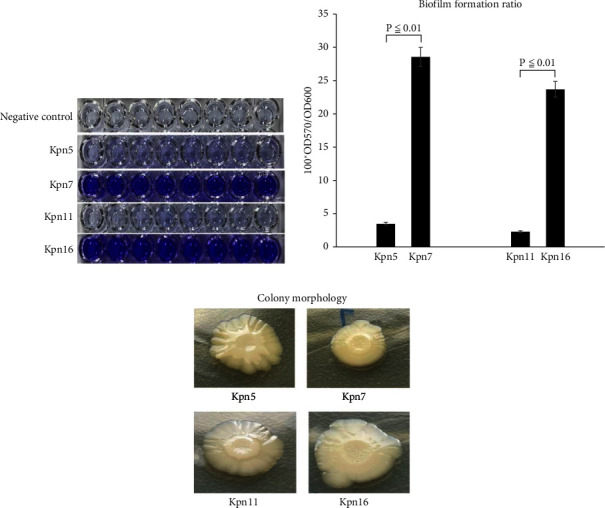
Differential analysis of biofilm formation by Kpn5, Kpn7, Kpn11, and Kpn16. (a) Biofilms formed on the 96-well plate, as demonstrated by crystal violet staining. (b) Quantification of biofilm formation by measuring OD_570_ and OD_600_ values. (c) Biofilm folds formed by the Kpn5, Kpn7, Kpn11, and Kpn16 strains on LB solid medium.

We set up LB solid media specially designed for the rugose colony morphology experiments and observed the phenotype after colony formation. The surfaces of the Kpn7 and Kpn16 colonies were relatively dry with obvious folds, but the Kpn5 and Kpn11 colony surfaces were smooth without obvious fold phenomena. Biofilm formation ratio determination showed that Kpn7 and Kpn16 formed more biofilms Figure 3(c). 96-well crystal violet staining assays and relative quantity of biofilm formation analysis (using OD_570_/OD_600_ values *P* ≤ 0.01) also showed that Kpn7 and Kpn16 formed relatively more biofilms (Figures 3(a) and 3(b)). Thus, these phenotypic experiments all indicated that the highly homologous strains had significant differences in biofilm formation abilities. Kpn7 and Kpn16 had much stronger biofilm formation capability than Kpn5 and Kpn11.

### 3.5. Expression Analysis of Types 1 and 3 Fimbriae of Four Experimental *ESBL-Producing K. pneumoniae* Strains

**Figure 4:**
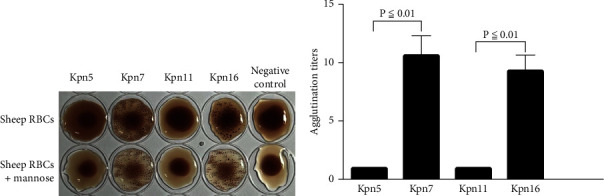
Sheep erythrocytes agglutination assay of ESBL-producing *K. pneumoniae* isolates. (a) Results of sheep erythrocyte agglutination by Kpn5, Kpn7, Kpn11, and Kpn16. Kpn7 and Kpn16 showed mannose-resistant agglutination reactions. (b) Sheep erythrocytes agglutination titers for Kpn5, Kpn7, Kpn11, and Kpn16.

**Figure 5:**
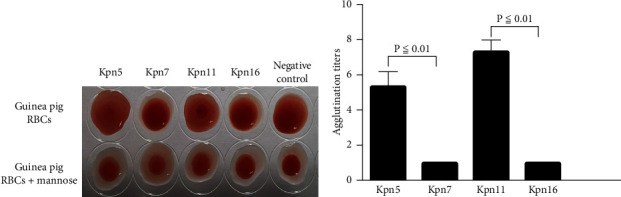
Guinea pig erythrocyte agglutination assay of ESBL-producing *K. pneumoniae* isolates. (a) Results of guinea pig erythrocyte agglutination by Kpn5, Kpn7, Kpn11, and Kpn16. Kpn5 and Kpn11 showed mannose-sensitive agglutination reactions. (b) Guinea pig erythrocyte agglutination titers for Kpn5, Kpn7, Kpn11, and Kpn16.

Most clinical ESBL-producing *K. pneumoniae* strains express both type 1 and type 3 fimbriae. Type 1 fimbriae can agglutinate guinea pig erythrocytes, but this reaction can be inhibited by D-mannose (“mannose-sensitive agglutination”). Type 3 fimbriae, in contrast, can agglutinate acidified sheep erythrocytes, but this reaction is not inhibited by D-mannose (“mannose-resistant agglutination”) [15]. Our results indicated that Kpn7 and Kpn16 showed mannose-resistant agglutination reactions in sheep erythrocytes, but Kpn5 and Kpn11 did not (Figure 4(a)). The sheep erythrocyte agglutination titer ratio for Kpn5 and Kpn7 was 1 : 10.07, but was 1 : 8.97 for Kpn11 and Kpn16 (Figure 4(b)). Additionally, guinea pig erythrocyte agglutination results showed that Kpn5 and Kpn11 had mannose-sensitive agglutination reactions, but Kpn7 and Kpn16 did not (Figure 5(a)). The guinea pig erythrocyte agglutination titer ratio for Kpn7 and Kpn5 was 1 : 5.03, but was 1 : 7.12 for Kpn16 and Kpn11 (Figure 5(b)). Taken together, these results suggest that Kpn7 and Kpn16 express more type 3 fimbriae than Kpn5 and Kpn11, and that overexpression of type 3 fimbriae is a major driver of the stronger biofilm formation abilities observed in the Kpn7 and Kpn16 strains. Interestingly, Kpn5 and Kpn11 expressed more type 1 fimbriae than Kpn7 and Kpn16.

### 3.6. RNA-Seq Transcriptomic Analysis

**Figure 6:**
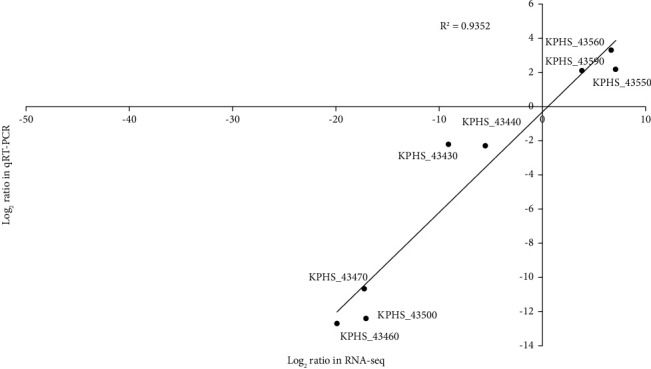
Comparison of transcription levels as determined by RNA-seq and quantitative reverse transcription-PCR. 8 genes were selected and subjected to qRT-PCR. The corresponding log2 values were plotted against RNA-seq data. The R^2^ for the two datasets was more than 0.90. R^2^: correlation coefficient.

RNA-seq was used to monitor differences in the transcriptomic mRNA profiles between Kpn5 and Kpn7 as well as Kpn11 and Kpn16 at a global scale. A total of 82 genes were found to be differentially expressed in both groups, with 26 of them having high fold differences (Log_2_^FoldChange^>2) (Table 4). After qRT-PCR verification, the correlation index reached 0.90, indicating that the sequencing data were reliable (Figure 6). *MrkABCDF* gene cluster expression was significantly upregulated in Kpn7 and Kpn16 compared to Kpn5 and Kpn11. Type 3 fimbriae are encoded by the *mrkABCDF* operon [16], which includes *mrkA,* which encodes the pilus subunit; *mrkB,* which helps control the organization of protein surface subunits; *mrkC,* which encodes the scaffold proteins for type 3 fimbriae and is located on the outer membrane; and *mrkD*, which encodes the tip of the fimbriae and endows it with its adhesive properties. *MrkF* can be randomly integrated into positively changing fimbriae to increase their stability [17–19]. We found that fimbriae 1 gene clusters were significantly upregulated in both Kpn5 and Kpn11 relative to Kpn7 and Kpn16. The mean log folds of the KPHS_43480, KPHS_43490, KPHS_43500, and KPHS_43510 gene cluster was 15.56 times higher in Kpn7 and Kpn16 than that in Kpn5 and Kpn11. Thus, this gene cluster may be related to differences in biofilm formation between these strains.

## 4. Discussion and Conclusion

### 4.1. Discussion

*K. pneumoniae* is a common opportunistic pathogen in hospitals. In 2017, the WHO classified ESBL-producing *K. pneumoniae* as a “critical threat” [20, 21]. Bacterial biofilms are known to play a significant role in chronic and device-associated infections and can render conventional antibacterial treatments ineffective [22–25]. In this study, we explored the homology and biofilm formation capacity of ESBL-producing *K. pneumoniae* isolates obtained from clinical specimens. Thirty clinical ESBL-producing *K. pneumoniae* isolates were collected from 25 patients aged an average of 62 years. Among these isolates, we found that 73% (22/30) could form biofilms. We also showed that 84% (21/25) of our included clinical patients had histories of surgery, urinary tract catheterization, and/or arteriovenous intubation, which can increase the risk of nosocomial opportunistic infections. The strains in this study were mainly drived from sputum (12, 41.3%), secretions (7, 24.1%), urine (5, 17.2%), blood (2, 6.8%), bile (2, 6.8%), and catheter (1, 3.4%). In comparison with isolates from different sources, the biofilm formation value was higher for isolates from sputum than from secretion, urine, blood, bile and catheter but the difference was not statistically significant. Instead, we found that high percentages of the isolates had the capacity to form biofilms *in vitro* but that strains that do not express type 3 fimbriae were not able to form biofilms. These data, although derived from a limited number of strains, are consistent with the results of previous studies. In one recent study, Hossein Ali Rahdar et al. reported that, among carbapenem-resistant *K. pneumoniae*, 77.9% (53/68) were able to form biofilms [23]. In another study, Vuotto et al. demonstrated that extensively ESBL-producing *K. pneumoniae* strains had strong biofilm generation capacities [26]. Binzhi Dan et al. reported the same results either [27]. Indeed, several studies have confirmed that biofilm capacity is associated with organisms viability in hospital environments, implanted medical devices, and patient wounds [28–30]. Numerous studies have shown that type 3 fimbriae are a critical factor in biofilm formation, especially within *K. pneumoniae* strains. This may be related to the ability of type 3 fimbriae to attach to the surface of *K. pneumoniae* and promote colonization and cloning [31–33].

The formation of bacterial biofilms has a significant impact on both patients and healthcare institutions, and understanding the mechanisms underlying biofilm formation may aid the development of more effective interventions. In our study, after PFGE cluster analysis, eight strains out of 30 clinical ESBL-producing *K. pneumoniae* isolates showed high homology and were suspected to have regional clonal transmission properties. 0.1% crystal violet staining experiments and bacterial fold results showed that the biofilm formation ability of the Kpn7 and Kpn16 strains far outstripped that of the Kpn5 and Kpn11 strains. One of the reasons for this difference may be related to the variability of bacterial living environments. Bacteria inside these biofilms needed to be able to quickly adapt to differing environmental stimuli and alter their gene expression. Both *K. pneumoniae* surface structures (fimbriae) and extracellular polysaccharides (cellulose) play an important role in biofilm formation [34, 35]. However, we did not detect cellulose production on our cellulase digestion assays or with *β*-1, 4-linkage specific calcofluor staining, findings which might be related to the low degree of cellulose synthesis that occurs in *in vitro*. Most of our clinical ESBL-producing *K. pneumoniae* isolates expressed type 1 and/or type 3 fimbriae, which we characterized, respectively, by their ability to mediate mannose-sensitive agglutination of guinea pig erythrocytes or their ability to mediate acidified sheep erythrocytes [36]. Findings from the acid-sheep and guinea pig RBC agglutination tests showed that the Kpn7 and Kpn16 strains expressed more type 3 and fewer type 1 fimbriae, but that the Kpn5 and Kpn11 expressed more type 1 and fewer type 3 fimbriae. Transcriptomic analysis showed that the mean log fold of the *mrkABCDF* gene cluster, which encodes type 3 fimbriae, was 12.01 times higher in the Kpn7 and Kpn16 strains than in the Kpn5 and Kpn11 strains. Additionally, the mean log fold of gene cluster encoding type 1 fimbriae was 4.66 times higher in the Kpn5 and Kpn11 strains than in the Kpn7 and Kpn16 strains. Phenotypic and molecular experiments indicated a positive correlation between biofilm formation ability and expression of type 3 fimbriae, and a negative correlation between the expression of type 1 fimbriae and biofilm formation. Di Martino et al. have also shown that the expression of type 3 fimbriae is positively correlated with biofilm formation abilities, but that strains only expressing the type 1 fimbriae cannot form biofilms [31]. Schroll et al.'s findings are also consistent with ours, as they found that biofilm formation in flow cell systems was associated with type 3 fimbriae rather than type 1 fimbriae, and that the expression of type 1 fimbriae was downregulated in *K. pneumoniae* strains which produced biofilms using a well-defined isogenic mutant [37]. Schroll et al. suggested that the downregulation of type 1 fimbriae in biofilm cells may be related to bacterial capsules, as the presence of capsules is crucial for the establishment and maturation of *K. pneumoniae* biofilms, and previous studies have confirmed that the presence of capsules could inhibit the functions of the type 1 fimbriae [38–40]. Nevertheless, the vast majority of *K. pneumoniae* isolates express both type 1 and type 3 fimbriae, and using epidemiological studies to characterize their role in biofilm formation is difficult.

The RNA sequencing-based transcriptome results showed that KPHS_43480, KPHS_43490, KPHS_43500, and KPHS_43510 gene cluster expression in the Kpn7 and Kpn16 strains was 15.56 times higher than that in the Kpn5 and Kpn11 strains. KPHS_43480 encodes proteins containing the DUF1471 domain, which has an unknown function, although it is present in several Enterobacteriaceae species (YdgH, YhcN, BhsA, and McbA) and may play a role in stress responses and/or biofilm formation and pathogenesis [41]. BhsA may decrease biofilm formation by repressing cell-cell interactions and cell surface interactions. YdgH and YhcN may respond to peroxide/acid stressors, and their paralogues have been implicated in bacteria pathogenesis. McbA may inhibit biofilm formation and overproduce colonic acid via currently unknown mechanisms. KPHS_43490 encodes EamA family transporters (formerly known as DUF6 transporters), which are part of several hypothetical membrane proteins of unknown functions [42]. KPHS_43500 encodes the MarR protein, which affects bacterial antibiotic resistance and the expression of capsule regulatory factors [43]. KPHS_43510 encodes a high-affinity nickel/cobalt transporter, which is involved in the incorporation of nickel into H2-uptake hydrogenase and urease and which is essential for the expression of catalytically active hydrogenase and urease [44]. Future studies should further characterize these regulatory mechanisms to better understand the processes underlying bacterial biofilm formation.

### 4.2. Conclusion

In this study, the genome homology and biofilm formation ability of 30 ESBL-producing *K. pneumoniae* strains from North Campus of Ruijin Hospital were initially explored. Even though two strains with the cut-off of PFGE reached 100% similarity, they generated biofilms differently. Meanwhile, high percentage of ESBL-producing *K. pneumoniae* isolates formed biofilms *in vitro*, and that strains that did not express type 3 fimbriae did not have biofilm formation capacity while type 1 fimbriae were downregulated during biofilm formation. Gene cluster of KPHS_43480, KPHS_43490, KPHS_43500, and KPHS_43510 may be related to biofilm, but few reported. Further studies were needed to fully understand the regulatory mechanisms of these genes.

## Figures and Tables

**Table 1:** Primers for the target genes used in qRT-PCR.

Gene name	Primers (5'-3')
KPHS_43560	CTGGCCGGTAATCATTGGAAC/GAAAGGGATTGGGTTGCTGTC
KPHS_43590	TTCTCGATCAGCAGCAGTCC/TGAAATTCCAGGGTGAATGTCG
KPHS_43550	GATTGTTGTGTCAGCCCTGTC/GCCGCATTAACGACTTCTCC
KPHS_43440	CAACATTAGCACCTCGTTCTCC/TGTAGCGGGTCTCCTGATTATTC
KPHS_43430	CGACTAACGATAATAATACTCTGGATAAG/ACATAGCCAACGTAATAGGTGAAC
KPHS_43470	CGGCAGCAGCGGATACTTAC/CTTCGGTGTTCGCCAGGTAG
KPHS_43460	GCCGTTCTACATTACCGTCAG/GCCACTTTCACTGCGACATC
KPHS_43500	TATTACCCGCCTGCCCAATC/TTCACCGTCACTGAGTCCTG
16 s	GAGCGGCGGACGGGTGAGTA/GGGCACATCTGATGGCATGA

**Table 2:** Characteristics of 30 antibiotic-resistant *K*. *pneumoniae* isolates.

Strain Id	Sex/Age	Inpatient ward	Sample type	Length of hospital stay (days)	Risk factors	Biofilm formation	Type 3 fimbriae
Kpn1	Man/81	ICU	Sputum	102	Urinary intubation/respirator	YES	±
Kpn2	Man/39	ICU	Sputum	21	Urinary intubation/arteriovenous cannula/respirator	YES	±
Kpn3	Man/56	ICU	Catheter	50	Arteriovenous cannula	YES	+++
Kpn4	Femal/63	ICU	Sputum	50	None	YES	++
Kpn5	Femal/55	Pulmonology	Urine	13	None	NO	—
Kpn6	Femal/69	ICU	Sputum	23	Urinary intubation/arteriovenous cannula/respirator	YES	±
Kpn7	Femal/45	Pulmonology	Urine	19	ICU	YES	+++
Kpn11	Man/30	ICU	Blood	28	Urinary intubation/arteriovenous cannula/respirator	NO	—
Kpn12	Man/65	ICU	Secretion	70	Surgery/urinary intubation/arteriovenous cannula/respirator	YES	+
Kpn13	Man/44	ICU	Bile	62	Surgery/urinary intubation/arteriovenous cannula/respirator	YES	+
Kpn14	Man/65	ICU	Bile	70	Surgery/urinary intubation/arteriovenous cannula/respirator	YES	+
Kpn15	Man/65	ICU	Blood	70	Urinary intubation/arteriovenous cannula/respirator	YES	+
Kpn16	Man/79	ICU	Secretion	52	Urinary intubation/arteriovenous cannula/respirator	YES	+++
Kpn21	Man/72	Emergency department	Urine	16	Urinary intubation/arteriovenous cannula/respirator	NO	—
Kpn22	Femal/63	ICU	Urine	50	Urinary intubation/arteriovenous cannula	YES	±
Kpn23	Man/60	Neurosurgery	Sputum	22	Urinary intubation/arteriovenous cannula/respirator	YES	+++
Kpn24	Femal/56	Neurosurgery	Sputum	21	Urinary intubation/arteriovenous cannula/respirator	YES	±
Kpn25	Man/61	Neurosurgery	Secretion	14	Surgery/urinary intubation/arteriovenous cannula/respirator	NO	—
Kpn31	Man/64	Hematology ward	Sputum	19	None	YES	±
Kpn32	Man/71	Orthopaedic	Secretion	35	Urinary intubation/arteriovenous cannula	YES	±
Kpn33	Man/71	ICU	Sputum	81	Urinary intubation/arteriovenous cannula/respirator	YES	±
Kpn34	Man/71	ICU	Secretion	81	Urinary intubation/arteriovenous cannula/respirator	YES	±
Kpn35	Man/34	Emergency department	Secretion	65	Arteriovenous cannula	YES	+
Kpn36	Femal/54	ICU	Secretion	17	Urinary intubation	NO	—
Kpn41	Man/76	ICU	Sputum	39	Urinary intubation/arteriovenous cannula/respirator	NO	—
Kpn42	Femal/51	ICU	Urine	43	None	YES	+
Kpn43	Man/39	Pulmonology	Sputum	42	Arteriovenous cannula	YES	+
Kpn44	Man/73	Pulmonology	Sputum	50	Urinary intubation/arteriovenous cannula/respirator	NO	—
Kpn45	Man/31	ICU	Sputum	19	Urinary intubation/respirator	YES	±
Kpn46	Man/67	Neurology	Sputum	23	None	NO	—

**Table 3:** Multidrug resistance spectrum of *K. pneumoniae* in this study.

Strain Id	Drug resistance spectrum	Number of resistant species
Kpn1	CPM-CAZ-CTX-AZM-CIP-AMS-MEM-SXT-GEN-LEV-PIP/SBT	11
Kpn2	CPM-CAZ-CTX-AZM-CIP-AMS-MEM-GEN-LEV-PIP/SBT	10
Kpn3	CPM-CAZ-CTX-AZM-CIP-AMS-IMI-MEM-SXT-GEN-LEV-PIP/SBT	12
Kpn4	CPM-CAZ-CTX-AZM-CIP-AMS-IMI-MEM-SXT-GEN-LEV-PIP/SBT	12
Kpn5	CPM-CAZ-CTX-AZM-CIP-AMS-IMI-MEM-SXT-GEN-LEV-PIP/SBT	12
Kpn6	CPM-CAZ-CTX-AZM-CIP-AMS-IMI-MEM-SXT-GEN-LEV-PIP/SBT	12
Kpn7	CPM-CAZ-CTX-AZM-CIP-AMS-IMI-MEM-SXT-GEN-LEV-PIP/SBT	12
Kpn11	CPM-CAZ-CTX-AZM-CIP-AMS-IMI-MEM-SXT-GEN-LEV-PIP/SBT	12
Kpn12	CPM-CAZ-CTX-AZM-CIP-AMS-IMI-MEM-SXT-GEN-LEV-PIP/SBT	12
Kpn13	CPM-CAZ-CTX-AZM-CIP-AMS-IMI-MEM-SXT-GEN-LEV-PIP/SBT	12
Kpn14	CPM-CAZ-CTX-AZM-CIP-AMS-IMI-MEM-SXT-GEN-LEV-PIP/SBT	12
Kpn15	CPM-CAZ-CTX-AZM-CIP-AMS-IMI-MEM-SXT-GEN-LEV-PIP/SBT	12
Kpn16	CPM-CAZ-CTX-AZM-CIP-AMS-IMI-MEM-SXT-GEN-LEV-PIP/SBT	12
Kpn21	CPM-CAZ-CTX-AZM-CIP-AMS-IMI-MEM-SXT-GEN-LEV-PIP/SBT	12
Kpn22	CPM-CAZ-CTX-AZM-CIP-AMS-IMI-MEM-SXT-GEN-LEV-PIP/SBT	12
Kpn23	CPM-CAZ-CTX-AZM-CIP-AMS-IMI-MEM-LEV-PIP/SBT	11
Kpn24	CPM-CAZ-CTX-AZM-CIP-AMS-IMI-MEM-SXT-GEN-LEV-PIP/SBT	12
Kpn25	CPM-CAZ-CTX-AZM-CIP-AMS-IMI-MEM-SXT-GEN-LEV-PIP/SBT	12
Kpn31	CPM-CAZ-CTX-AZM-CIP-AMS-IMI-MEM-SXT-GEN-LEV-PIP/SBT	12
Kpn32	CPM-CAZ-CTX-AZM-CIP-AMS-IMI-MEM-SXT-GEN-LEV-PIP/SBT	12
Kpn33	CPM-CAZ-CTX-AZM-CIP-AMS-IMI-MEM-SXT-LEV-PIP/SBT	11
Kpn34	CPM-CAZ-CTX-AZM-CIP-AMS-IMI-MEM-SXT-LEV-PIP/SBT	11
Kpn35	CPM-CAZ-CTX-AZM-CIP-AMS-IMI-MEM-SXT-GEN-LEV-PIP/SBT	12
Kpn36	CPM-CAZ-CTX-AZM-CIP-AMS-IMI-MEM-SXT-GEN-LEV-PIP/SBT	12
Kpn41	CPM-CAZ-CTX-AZM-CIP-AMS-IMI-MEM-SXT-LEV-PIP/SBT	11
Kpn42	CPM-CAZ-CTX-AZM-CIP-AMS-IMI-MEM-SXT-GEN-LEV-PIP/SBT	12
Kpn43	CPM-CAZ-CTX-AZM-CIP-AMS-IMI-MEM-SXT-GEN-LEV-PIP/SBT	12
Kpn44	CPM-CAZ-CTX-AZM-CIP-AMS-IMI-MEM-SXT-GEN-LEV-PIP/SBT	12
Kpn45	CPM-CAZ-CTX-AZM-CIP-AMS-IMI-MEM-SXT-GEN-LEV-PIP/SBT	12
Kpn46	CPM-CAZ-CTX-AZM-CIP-AMS-IMI-MEM-SXT-GEN-LEV-PIP/SBT	12

**Table 4:** List of 26 significantly differentially expressed genes in Kpn5 VS Kpn7 and Kpn11 VS Kpn16.

Gene Id	MeanTPM (Kpn5/Kpn7)	MeanTPM (Kpn11/Kpn16)	Result	Log_2_^FoldChange^	Gene description
KPHS_43430	1.87/72.58	1.5/87.51	Down	−5.3/−5.9	Putative fimbrial-like protein
KPHS_43440	0.21/136.92	0.34/164.2	Down	−9.3/−8.9	Putative fimbrial-like protein
KPHS_43450	0.39/115.66	0.48/135	Down	−8.2/−8.1	Putative fimbrial usher protein
KPHS_43460	0.0001/86.42	0.0001/116.81	Down	−19.7/−20.1	MrkB fimbrial protein
KPHS_43470	0.0001/1383.18	0.86/1622.43	Down	−23.7/−10.9	MrkA fimbrial protein
KPHS_43480	0.0001/15.31	0.0001/38.12	Down	−17.2/−18.5	Hypothetical protein
KPHS_43490	0.0001/0.4	0.0001/0.31	Down	−11.9/−11.6	EamA family transporter
KPHS_43500	0.0001/12.61	0.0001/16.53	Down	−16.9/−17.3	Putative regulatory protein MarR
KPHS_43510	0.0001/0.86	0.0001/1.57	Down	−13.1/−13.9	Nickel/cobalt transporter
KPHS_43550	338.41/3.97	639.63/3.09	Up	6.4/7.69	Major type 1 subunit fimbrin (pilin)
KPHS_43560	27.4/0.53	65.83/0.35	Up	5.7/7.6	Fimbrial protein involved in type 1 pilus biosynthesis
KPHS_43570	23.96/0.2	25.75/0.46	Up	6.9/5.8	Periplasmic chaperone
KPHS_43580	9.36/0.69	17.01/0.71	Up	3.8/4.6	Outer membrane protein for export and assembly of type 1 fimbriae
KPHS_43590	25.75/1.69	40.39/3.12	Up	3.9/3.7	Type 1 fimbrial minor component
KPHS_43600	11.82/1.03	28.91/2.42	Up	3.5/3.6	Fimbrial morphology protein
KPHS_43610	14.34/1.61	28.08/2.07	Up	3.2/3.2	Fimbrial protein FimH
KPHS_43620	3.76/0.81	10.34/1.36	Up	2.2/2.9	Putative fimbrial protein
KPHS_47790	6.35/0.91	3.02/1.02	Up	2.8/1.6	Hypothetical protein
KPHS_35550	56.19/7.41	42.15/3.9	Up	2.9/3.4	dTDP-4-dehydrorhamnose 3,5-epimerase
KPHS_33690	40.52/6.7	13/4.17	Up	2.6/1.6	Hypothetical protein
KPHS_12940	9.41/1.51	4.29/1.41	Up	2.6/1.6	Hypothetical protein
KPHS_04400	30.47/2.2	5.14/2.39	Up	3.8/1.1	Hypothetical protein
KPHS_35580	47.58/11.78	36.66/7.91	Up	2.0/2.2	dTDP-D-glucose 4,6-dehydratase
KPHS_35570	46.72/11.01	41.52/7.15	Up	2.1/2.5	Glucose-1-phosphate thymidylyltransferase
KPHS_31660	40.98/9.19	29.51/11.04	Up	2.2/1.4	Putative translation initiation inhibitor
KPHS_17580	17.19/3.82	29.47/11.84	Up	2.2/1.3	Oxygen-insensitive NADPH nitroreductase

## Data Availability

All data generated or analysed during this study are included in this manuscript. The RNA-seq data have been submitted in National Center for Biotechnology Information (BioProject: PRJNA1132956) (SRA:SRR29734451/SRR29734452/SRR29734453/SRR29734454) (https://www.ncbi.nlm.nih.gov/sra?LinkName=bioproject_sra_all&from_uid=1132956). The data that support the findings of this study are available from the corresponding authors upon reasonable request.
